# Sentence-final completion norms for 2925 Mexican Spanish sentence contexts

**DOI:** 10.3758/s13428-023-02160-y

**Published:** 2023-07-05

**Authors:** Armando Quetzalcóatl Angulo-Chavira, Alejandra Mitzi Castellón-Flores, Alejandra Ciria, Natalia Arias-Trejo

**Affiliations:** Facultad de Psicología, Universidad Nacional Autónoma de México, Mexico City, Mexico

**Keywords:** Sentence-ending norms, Cloze probability, Surprisal, Entropy, Sentence language processing

## Abstract

Sentence-final completion tasks serve as valuable tools in studying language processing and the associated predictive mechanisms. There are several established sentence-completion norms for languages like English, Portuguese, French, and Spanish, each tailored to the language it was designed for and evaluated in. Yet, cultural variations among native speakers of the same language complicate the claim of a universal application of these norms. In this study, we developed a corpus of 2925 sentence-completion norms specifically for Mexican Spanish. This corpus is distinctive for several reasons: Firstly, it is the most comprehensive set of sentence-completion norms for Mexican Spanish to date. Secondly, it offers a substantial range of experimental stimuli with considerable variability in terms of the predictability of word sentence completion (cloze probability/surprisal) and the level of uncertainty inherent in the sentence context (entropy). Thirdly, the syntactic complexity of the sentences in the corpus is varied, as are the characteristics of the final word nouns (including aspects of concreteness/abstractness, length, and frequency). This paper details the generation of the sentence contexts, explains the methodology employed for data collection from a total of 1470 participants, and outlines the approach to data analysis for the establishment of sentence-completion norms. These norms provide a significant contribution to fields such as linguistics, cognitive science, and machine learning, among others, by enhancing our understanding of language, predictive mechanisms, knowledge representation, and context representation. The collected data is accessible through the Open Science Framework (OSF) at the following link: https://osf.io/js359/?view_only=bb1b328d37d643df903ed69bb2405ac0.

## Introduction

The primary function of a listener is to decipher the meaning of a speaker's message. However, understanding such a message transcends merely aggregating the meanings of individual words. The interplay among words in a message aids in deciphering the meaning of individual words. To illustrate, to disambiguate the meaning of the word *bat*, the semantic context of the sentence must determine whether *bat* refers to an animal or a piece of sports equipment. Therefore, as the sentence context unfolds, the suitable meaning is chosen, and unsuitable meanings are disregarded (Stanoyich & West, 1983).

The sentence context proves invaluable in formulating a prediction for the forthcoming word, thus facilitating faster processing and lessening the cognitive effort of bottom-up processing (Huettig, 2015; Kuperberg & Jaeger, 2016; Pickering & Gambi, 2018). For instance, in the sentence, *The day was breezy, so the boy went outside to fly a...*, the context strongly hints at the word *kite* being the likely completion. Conversely, in the sentence, *Wally wanted to buy a beer, but he was too...*, the context does not strongly suggest *young* as the completing word. This less-constrained sentence context also accommodates other possibilities such as *broke*, *drunk*, *poor*, *cheap*, *full*, or *lazy*. Evidence suggests that a highly constrained sentence context can hasten response times in a word production task (Staub et al., 2015), streamline reading (Smith & Levy, 2013), and reduce the neural resources needed for reading comprehension (DeLong et al., 2005).

Sentence context constraint is not a binary, but rather a continuous function, typically gauged by the cloze procedure (Taylor, 1953). In this procedure, a word is removed from a sentence, and participants are asked to complete the sentence by filling in the gap with the most suitable word according to the sentence context. The participant-provided words from the cloze procedure are then used to calculate the cloze probability, which is the percentage of participants who filled in the sentence with the same word (Bloom & Fischler, 1980). Cloze probability is strongly correlated with behavioral and electrophysiological measures (DeLong et al., 2005; Smith & Levy, 2013; Staub et al., 2015). For instance, the N400 component is larger for less predictable words than for more predictable ones, and its amplitude is strongly related to the cloze probability (with correlation coefficients as high as .84; e.g., DeLong et al., 2005). This inverse correlation indicates that a larger N400 amplitude corresponds to a lower cloze probability value, suggesting that more predictable words require fewer neural resources.

Research indicates that the probability of words does not follow a linear function but is instead logarithmic (Smith & Levy, 2013). This logarithmic relationship implies that a small variation in cloze probability can lead to significant changes in behavioral (e.g., reaction or reading times) or physiological (e.g., N400 component, BOLD response) measures (Yan et al., 2017). Therefore, surprisal has been proposed as a measure of the probability of words in a sentence context (Levy, 2008), as it aligns more closely with the logarithmic nature of language prediction. Surprisal is defined as the negative logarithm of the conditional probability of a word, given a preceding sentence context (Levy, 2008). According to Shannon's information theory (Shannon, 1948), surprisal can be conceptualized as the quantity of information gained when a word is processed in the context of a sentence. Thus, a low-probability word generates higher surprisal values because it adds more information to the overall meaning of the sentence. On the other hand, a high-probability word produces less surprisal because it is anticipated, and therefore, it does not add any new information.

Similar to cloze probability, surprisal also correlates with behavioral and electrophysiological measures such as reading times or the N400 component (Frank et al., 2015; Smith & Levy, 2013). However, some studies have shown that surprisal, but not cloze probability, is associated with the N400 component elicited during language processing (Delaney-Busch et al., 2017; Yan et al., 2017). Furthermore, since surprisal is interpreted as the information gained by incorporating a new word into the sentence context, this measure aligns more closely with the predictive processing framework. This theoretical model posits that our predictions about forthcoming words are consistently tweaked and refined based on the received linguistic information. In other words, when we encounter unexpected or surprising linguistic input, it prompts a significant update in our predictions. This is because unexpected information necessitates a larger prediction adjustment, enabling us to make better predictions in the future. Conversely, if the input is anticipated or less surprising, only minor changes are required in our predictive process, as the new word aligns with our previous expectations (Kuperberg & Jaeger, 2016).

Given that the probability of a word depends on the sentence context, it is possible to quantify the uncertainty of a sentence’s context using entropy (Lowder et al., 2018). Entropy in a sentence context is a function of the probability of all potential words that could continue the sentence at different target points. Sentences with high entropy tend to have equally probable word continuations at a given target point in the sentence. As such, a sentence with high entropy activates multiple possible words in memory due to the uncertainty of which of the equally probable words will follow (Elman et al., 2004). In contrast, sentences with low entropy often show a significant difference in the probability of continuations at a particular target point. Such a sentence with low entropy facilitates the activation of the most probable word continuation. For instance, consider the sentence *When he got out of the car, he closed the…* which has two possible continuations: *door* and *window*, with cloze probabilities of .98 and .02, respectively. On the other hand, the sentence *In the night, the woman closed the…* has the same two possible continuations, *door* and *window*, but with nearly equal cloze probabilities, .55 and .45, respectively. Based on the distribution of cloze probabilities and the number of potential word continuations in both sentences, the first sentence has low entropy (.15), while the second has high entropy (.99).

Cloze probability, surprisal, and entropy are metrics that can be computed from sentence-ending norms. Such norms evaluate multiple sentences with groups of participants using the cloze procedure, thus providing a reliable measure of the constraints within sentences and their possible endings for experimental psycholinguistic studies. As of now, sentence-final completion norms are available for various languages including English (Bloom & Fischler, 1980; Peelle et al., 2020), French (Robichon et al., 1985), Portuguese (Pinheiro et al., 2010; Rossi et al., 2020), and Spanish (McDonald & Tamariz, 2002; Rodríguez-Camacho et al., 2011). These norms serve as valuable resources for psycholinguistic research intended to enhance our understanding of the cognitive processes involved in language processing, and to study the brain regions involved (DeLong et al., 2021; Federmeier et al., 2002; Kutas & Hillyard, 1984; Wang et al., 2018). Moreover, sentence-ending norms are beneficial for studying clinical populations with memory- and language-related issues (Pinheiro et al., 2010; Wolk et al., 2009; Ye et al., 2021).

The primary objective of the present research is to offer a comprehensive set of sentence-completion norms in Mexican Spanish. This will contribute a broad range of experimental stimuli for psycholinguistic research and other cognitive science studies that require such stimuli. While past research has provided a large set of sentence-completion norms in English (Peelle et al., 2020), comparable norms in Spanish are still lacking. Syntactic rules of a language, along with its phonological and morphological differences, can affect the predictability of words. Spanish, for instance, is a gender and number-marked language; thus, articles often provide information about the gender (masculine/feminine) and quantity (singular, plural) of the forthcoming word. For instance, in English, the article *the* provides information about the syntactic class of the upcoming word, whereas, in Spanish, the articles *el*/*la*/*los*/*las* (equivalent to *the* in English) provide additional information about gender and number.

To the best of our knowledge, there exist only two norms for Spanish speakers (McDonald & Tamariz, 2002; Rodríguez-Camacho et al., 2011). Despite their value, these Spanish norms present certain limitations. Firstly, both Spanish norms offer a small set of sentences, 112 and 278, respectively. Secondly, the cloze probability of the sentences in both norms is not well balanced across different levels, complicating stimulus selection. For example, electrophysiological studies often require a minimum of 80 trials per condition to achieve reliable measures (Duncan et al., 2009). In a hypothetical study comparing high (> .67) versus low (< .30) cloze probability sentences, only 30 sentences would meet the high constraint criterion, and 32 would meet the low constraint criterion in McDonald and Tamariz's (2002) norms. In contrast, Rodríguez-Camacho et al.'s (2011) norms would present 127 high-constrained and 37 low-constrained sentences. Thus, unless the experimental design involved the use of repeated stimuli, both norms would fall short of meeting the minimum number of trials required for physiological research.

Moreover, McDonald and Tamariz's (2002) norms were evaluated for native Spanish speakers from Spain. However, due to cultural variations among native speakers of the same language, it is challenging to assert the universality of these norms (Arcuri et al., 2001; Rossi et al., 2020). For instance, Arcuri et al. (2001) identified differences in the cloze probability between English speakers from the United States and England. Particularly relevant to this work, some dialectical variants between Mexican Spanish and Spanish from Spain can impact the cloze probabilities of the sentences evaluated for establishing norms. Notable dialectical differences include pronominal systems (e.g., Spain: *vosotros* vs. Mexico: *ustedes* [*you*]), phonetics (e.g., in Spanish from Spain, the phonemes s, c, z are well differentiated when pronounced compared to Spanish from Mexico), frequent syntactic constructions (Spain: *me has pedido que me vaya* vs. Mexico: *me pediste que me vaya* [*you ask me to leave*]), and lexical usage (e.g., Spain: *ordenador* vs. Mexico: *computadora* [*computer*]). These differences can influence cloze probability (Arcuri et al., 2001; Rossi et al., 2020), especially in low-probability sentences (Rossi et al., 2020).

By contrast, Rodríguez-Camacho et al.'s (2011) norms were assessed by children (9–12 years old) who were native Spanish speakers from Mexico. The cloze probability of these sentences may not be applicable when used with adults as the sentence contexts are primarily based on children's experiences and syntactic knowledge. Furthermore, previous studies have shown that sentence norms vary across ages in terms of response variability because children provide more idiosyncratic and invalid responses (Pinheiro et al., 2010; Rossi et al., 2020). The differences in vocabulary size and frequency of use between children and adults can also lead to differences in sentence-completion tasks. Children’s responses primarily affect the measure of entropy due to the number of responses, and to a lesser extent, they also impact the measures of surprisal and cloze probability.

Considering the previous evidence, the main objective of this work is to provide a large set of sentence-completion norms in Spanish from adult Mexican speakers, aiming to promote research into the role of prediction in language processing. A corpus of 2925 sentence-completion norms for Mexican Spanish has been created. The sentences feature a high degree of variability in the predictability of word sentence completion (cloze probability/surprisal) and the level of uncertainty of the sentence context (entropy). Moreover, the sentences display diversity in syntactic complexity and the properties of the final word nouns (concreteness/abstractness, length, frequency). As far as we are aware, this is the most extensive set of sentence-completion norms in Spanish from Mexico to date.

## Method

### Participants

We recruited participants for this study in 2020 and 2021 by posting advertisements on the social media networks of various universities across Mexico. These advertisements contained a link and a QR code that directed interested individuals to the online experimental task. A total of 1524 participants were included in this research. However, 54 participants were later excluded for a variety of reasons including not speaking Spanish as their first language (*n* = 3), not being Mexican (*n* = 3), being younger than 18 years old (*n* = 5), providing responses that were nonsense words or letters (*n* = 10), or only completing the demographic information but not the cloze task (*n* = 34). This resulted in a final sample of 1470 adults with an average age of 25.78 years (*SD* = 5.34; *range* = 18–57.21). The gender distribution of the participants was 75.57% women (*n* = 1111), 23.26% men (*n* = 342), and 1.15% (*n* = 17) nonbinary.

Additional demographic and personal information were collected from each participant, such as education level, history of any developmental, psychiatric, or neurological disorders, and any drug use or medications. Participants were also asked about their knowledge of other languages, the age at which they acquired these languages, and their self-rated proficiency in speaking, listening, writing, and reading in those languages. The purpose of collecting these additional variables was to provide a comprehensive description of the participant sample for future studies. By doing so, researchers who use these norms can select data from participants whose profiles align with their specific experimental research questions.

In terms of geographical representation, participants hailed from all 32 states in Mexico, with the largest proportion, 69.18% (*n* = 1,017), from the South-Central Region. This was followed by 7.34% (*n* = 108) from the Northwest Region, 7.14% (*n* = 105) from the East Region, 6.93% (*n* = 102) from the West Region, 3.60% (*n* = 53) from the North-Central Region, 2.38% (*n* = 35) from the Southeastern Region, 1.76% (*n* = 26) from the Southwestern Region, and 1.63% (*n* = 24) from the Northeast Region. Most participants reported living in their current state of residence since birth, with an average duration of residence of 21.30 years (SD = 8.27; range, 0.02–54).

In relation to education, 44.76% of participants had completed an undergraduate degree (*n* = 658), 47.27% a graduate degree (*n* = 695), 6.87% a master's degree (*n* = 101), and 1.08% a PhD (*n* = 16). When asked about their field of study (which was an optional question), 44.89% reported studying social sciences and humanities (*n* = 660), 25.64% biological and medical sciences (*n* = 377), 9.37% physical and mathematical sciences or engineering (*n* = 137), and 5.37% economics and administrative sciences (*n* = 79).

A small proportion of participants reported a diagnosis of developmental disorders, including attention deficit hyperactivity disorder (4.69%, *n* = 69), dyslexia (2.72%, *n* = 40), autistic spectrum (0.7%, *n* = 11), language delay (0.7%, *n* = 11), motor delay (0.6%, *n* = 1), intellectual disability (0.6%, *n* = 1), and dyscalculia (0.2%, *n* = 4).

Regarding psychiatric disorders, 24.48% of participants reported a diagnosis of anxiety (*n* = 360), followed by depression (19.11%, *n* = 281), obsessive–compulsive disorder (2.99%, *n* = 44), post-traumatic stress disorder (2.44%, *n* = 36), eating disorders (1.83%, *n* = 27), drug dependence (1.49%, *n* = 22), bipolar disorder (0.68%, *n* = 10), dissociative disorders (0.54%, *n* = 8), and schizophrenia (0.27%, *n* = 4). A few participants reported history of neurological conditions such as epilepsy (0.95%, *n* = 14), brain trauma (0.47%, *n* = 10), and stroke (0.34%, *n* = 5). Some participants reported using substances such as antidepressants (6.53%, *n* = 96), anxiolytics (3.40%, *n* = 50), opiates (1.83%, *n* = 27), antipsychotics (0.68%, *n* = 10), and mood stabilizers (0.40%). Additionally, 2.78% (*n* = 41) admitted to using an illegal drug within the previous 24 h.

Almost half of the participants (48.50%) reported speaking at least one language other than Mexican Spanish, with a total of 12 other languages reported. On average, these participants reported acquiring a second language at 11.13 years old and being exposed to it for an average of 5.43 hours per week. They reported an average middle-low level of proficiency in their second language, based on a 0–4 scale (Speaking: *M* = 1.54, *SD* = 0.80; Listening: *M* = 1.09, *SD* = 0.82; Writing: *M* = 1.30, *SD* = 0.79; Reading: *M* = 0.77, *SD* = 0.77). A tiny fraction, 0.01% (*n* = 21), reported speaking more than two languages.

#### Generation of sentence contexts

The set of sentences used in this study was comprised of a total of 2925 sentence contexts. 1563 of these sentences were sourced from existing norms in various languages (Block & Baldwin, 2010; Bloom & Fischler, 1980; Brothers & Kuperberg, 2021; Lahar et al., 2004; Nieuwland et al., 2020; Wang et al., 2018), including two previously established Spanish norms (McDonald & Tamariz, 2002; Rodríguez-Camacho et al., 2011). Norms from other languages were translated into Spanish and adapted to a Mexican context.

Inclusion of sentence contexts from these norms was based on the feasibility of straightforward translation or adaptation for adult Mexican Spanish speakers. Sentence contexts highly focused on children from Rodríguez-Camacho et al.’s (2011) norms, for instance, were excluded. Also, sentence contexts with strong cultural associations (e.g., Thanksgiving), sayings (e.g., *the ball is in your court*), or sentences where the expected word was a derivative of another word in the sentence (e.g., *panadería* – *pan* [*bakery - bread*]) were omitted. All expected words were nouns and were placed at the end of the sentence when necessary.

In the end, 1263 sentences were selected, which did not include an additional set of 109 sentences initially selected from these norms but were discarded due to a significant loss of their main meaning during the adaptation process. From these 1263 sentences, we derived the words with the highest cloze probability (*n* = 670). Given that most nouns only had one or two levels of constraint according to the original norms, our research group created 1672 additional sentences to ensure a minimum of three sentences per noun with varying levels of cloze probability: high (> .70), medium (.40–.70), and low (< .40).

The newly created sentences, not derived from previous studies, were carefully crafted to adhere to certain criteria. Firstly, we ensured these sentences were both plausible and grammatically correct in their contextual meanings within Mexican Spanish. This meant the sentences were easily understandable to native speakers and effectively communicated the intended meaning. A crucial element of these sentences was the positioning of the critical word, which was always at the end of the sentence. The average length of these sentences was kept to about eight words.

To set the putative level of constraint, we utilized the "perceived entropy" of the research group. The putative level of cloze probability was determined by the number of words that could logically complete the sentence. The following were the criteria used:

For sentences with high constraint levels, we created them using semantically informative verbs, nouns, adjectives, and contexts to facilitate the elicitation of the expected word. For instance, consider the sentence: *En invierno mi abuelita me tejió un…* [*In winter, my grandmother knitted me a*]. In this example, the nouns *invierno* [*winter*] and *abuelita* [*grandmother*] set a suitable context for the word *suéter* [*sweater*], in conjunction with the verb *tejer* [*knit*]. Additionally, we accounted for Spanish gender and number marking; for example, the use of the article *un* [*a*] limited the lexical candidates to a singular masculine noun. This is also applicable for *pluralia tantum*, e.g., *tijeras* [*scissors*]. To further ensure accuracy, we avoided the use of morphologically derived nouns related to the target word, like using *panadería* [*bakery*] as a clue for *pan* [*bread*], or *carnicería* [*butcher shop*] for *carne* [*meat*].

Medium constraint-level sentences were crafted by choosing nouns and adjectives associated with a limited set of referents that share the same semantic context invoked by the sentence. An example is: *Hacía frío, entonces regresé por mi*… [*It was so cold that I came back for my*], which could elicit responses like *chaqueta* [*jacket*], *bufanda* [*scarf*], *suéter* [*sweater*], *abrigo* [*coat*], or *sudadera* [*sweatshirt*], without dictating a single correct answer. Moreover, the use of the possessive pronoun *mi* [*my*] avoids any gender and number marking.

Sentences with low probability levels were created using semantically non-informative verbs, nouns, contexts, and adjectives. This approach allowed for a broad range of potential sentence completions, although one option was usually slightly more probable. An example of such a sentence is: *En invierno fui al centro comercial a comprar un*… [*In winter, I went to the mall to buy a*], which could be completed with a wide variety of items, including *suéter* [*sweater*], *vino* [*wine*], *celular* [*cellphone*], *abrigo* [*coat*], *videojuego* [*videogame*], *vestido* [*dress*], *pelota* [*ball*], or *pastel* [*cake*], among others.

As a result, the final set of novel sentence contexts had a putative cloze probability distributed as follows: 1266 high, 741 medium, and 917 low. All sentences were syntactically and grammatically correct. The sentence contexts had a median length of eight words, ranging from 5 to 13 words. This length variability allowed for a broad range of syntactic structures, including simple, coordinate, or subordinate sentences, and transitive or intransitive verbs.

A syntactic analysis of the corpus revealed that it included 864 independent clauses (including those with subordination, coordination, or juxtaposition) and 1626 simple sentences (including transitive, intransitive, or quasi-reflex). Additionally, 289 subordinate clauses were identified. The morphology of each sentence was reviewed using Freeling (Padro & Stanilovsky, 2012) to determine the grammatical category and morphological composition of each word.

#### Procedure

The cloze procedure was conducted using Cognition (https://www.cognition.run/) via jsPsych (version 6.3.0), a JavaScript framework for creating experiments that can run in a web browser (de Leeuw, 2015). The task was designed to allow participants to respond using their preferred electronic device, be it a computer, tablet, or cellphone. Initially, participants were asked to read the informed consent document. If they did not agree with the terms of the experiment, the platform automatically redirected them to Cognition's homepage. Upon agreement, the experiment commenced with demographic questions. Following this, participants were shown the instructions for the task (Appendix), as well as a practice trial that featured three examples not included in the main experiment. Participants could repeat this practice trial as often as needed. Next, the cloze task was initiated. Participants were tasked with writing the word that best completed each sentence context as quickly as possible. A time limit of 15 s was imposed for each response. If participants failed to provide a response within this time frame, they were automatically moved to the next sentence. A total of 2925 sentences were tested, divided into 25 lists of 117 sentences each. The specific list assigned to each participant was chosen at random from the set of lists, with an equal probability specified for each list to ensure controlled selection. Upon selection, the order of sentences within each list was randomized for every participant. Participants could choose to answer as many or as few of the sentences as they wished. As a result, participants' data were included even if they had only responded to a small proportion of the experimental list. Out of the 1470 total participants, 1302 (88.57%) completed all 117 sentences, with an average of 110.86 sentences completed (SD = 21.57, range, 1–117).

#### Data analysis

Before beginning the formal analysis, we manually corrected several aspects of the responses. We corrected any misspelled words and converted all words to lowercase. We removed any extra spaces. If there were responses that varied in tense, number, grammatical gender, or synonyms, we combined them where appropriate. Any empty responses, unrecognizable words or non-existent words were deemed errors and replaced with NA. These unified data were used for all analyses. However, both the raw data and the orthographically corrected data are stored in the OSF repository.

We carried out all descriptive analyses in MATLAB. For the responses to the Experimental task, we computed three metrics: cloze probability, surprisal, and entropy. The method we used to calculate cloze probability was as follows:

$${p(cloze)}_{i}= \frac{f({x}_{i})}{{\sum }_{i=1}^{N}{f(x}_{i})}$$

In our analysis, we denote *f* as the absolute frequency for the *i*-th word *x*. The cloze probability is calculated by adding up the repetitions of the same word and then dividing this total by the number of total responses. It is important to note that each different response has its own unique cloze probability. We used the following method to compute surprisal:

$$surprisal= {-log}_{2}\left(\frac{f\left({x}_{i}\right)}{{\sum }_{i=1}^{N}{f(x}_{i})}\right)$$

Following Shannon's information theory (Shannon, 1948), we used base two logarithms to interpret surprise. The idea is to represent surprise as the number of 'bits' of information that are gained when the final word in the sentence is encountered. We then proceeded to compute the entropy of a word using the following methodology:

$$entropy= -\sum\limits_{i=1}^{N}\left(\frac{f\left({x}_{i}\right)}{{\sum }_{i=1}^{N}{f(x}_{i})}\right)\left({log}_{2}\left(\frac{f\left({x}_{i}\right)}{{\sum }_{i=1}^{N}{f(x}_{i})}\right)\right)$$

In keeping with the calculation of surprisal, we used base two logarithms in the computation of entropy values. This approach aligns with Shannon's information theory (Shannon, 1948), enabling us to interpret entropy values within this theoretical framework.

Pearson correlations were performed to ascertain if the measures align with previous research involving cloze probability, surprise, entropy, reaction times, invalid responses, and number of responses. All measures were expected to be highly correlated, albeit in varying directions. We specifically anticipated that constraining measures (cloze probability, surprise, and entropy) would modulate reaction times, the number of responses, and the number of invalid responses.

We also explored participant variability based on their demographic data, specifically looking at how factors such as gender, region of residence, educational background, field of study, neuropsychological profile, or knowledge of a second language might affect our predictability measures (cloze probability, surprise, entropy) and reaction times. As not all levels of each demographic variable had sufficient participants, we created binary groups based on participant numbers. Thus, comparisons were made between women and men in terms of gender; south-center versus other regions of the country in terms of residence; undergraduate versus graduate in terms of education; social sciences and humanities versus all other fields in terms of academic discipline; neurotypical individuals versus those who reported any neurodevelopmental, neuropsychiatric, or neurological diagnosis; and monolinguals versus individuals who reported knowledge of other languages.

Although we aimed to create evenly distributed groups, participants were not evenly distributed across all sentences. Consequently, we utilized univariate ANCOVAs for data comparison, using the grouping variable as a fixed factor and the number of participants who answered each sentence as a covariate.

Lastly, we contrasted cloze probabilities from previous studies’ corpus in three main analyses. The first comparison involved the Rodriguez‐Camacho et al. (2011) children’s norms and our data to assess the effect of development on Mexican Spanish speakers. The second comparison utilized the McDonald & Tamariz (2002) Spanish from Spain’s norms to evaluate the effect of dialectal variation on cloze probability. The third comparison involved norms in languages other than Spanish and our own data, to examine the effect of translation and adaptation on cloze probabilities. For all three comparisons, we only included a sentence in the analysis if the modification did not alter the general meaning of the sentences and if the intended final completion was different between norms. We then compared cloze probabilities using paired *t* tests to mitigate the variability introduced by all levels of cloze probability.

## Data availability statement

All data, including the calculated measures for each sentence and the demographic information of the participants, are freely available on the Open Science Framework repository at the following link: https://osf.io/js359/?view_only=bb1b328d37d643df903ed69bb2405ac0. We have also provided a MATLAB script that can be used to compute cloze probability, surprisal, and entropy based either on the complete dataset or by excluding participants according to their demographic information.

## Results

### General measures

Each sentence was evaluated by an average of ~ 55 participants (*SD* = 6.84, *range* = 38–68) who provided a response in the sentence-completion task. Of the total responses, 157,815 (96.83%) were valid words, while 5171 (3.17%) were incomplete words or empty responses (invalid). In the database, all the invalid responses were classified as NA. The valid responses consisted of 6720 different words, with a median frequency of three repetitions per word (*mode* = 1; *range* = 1–1558 repetitions). A total of 2421 different responses had a frequency of 1, representing 36.02% of the total unique responses (6720); this result indicated that approximately a third of the responses in the corpus are idiosyncratic responses. Each unique word thus appeared in a median of two sentences (*mode* = 2; *range* = 1–238). This distribution remained relatively consistent even when the idiosyncratic responses were excluded (*median* = 3; *mode* = 2, *range* = 1–238); suggesting that approximately half of the word responses are included in at least three different sentences.

**Fig. 1:**
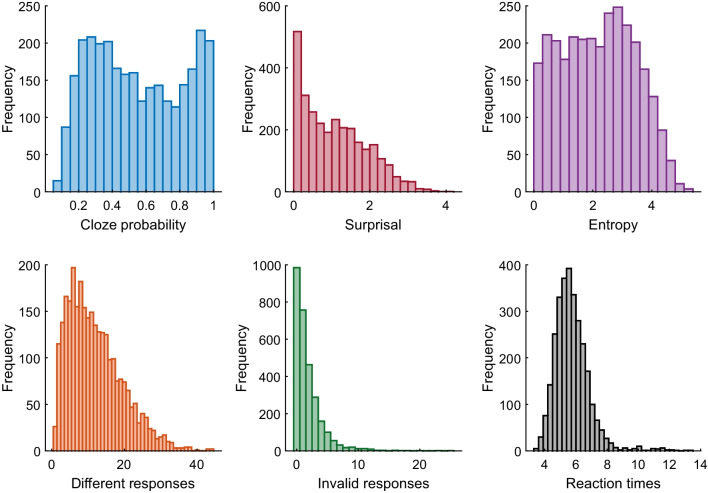
Distribution of measures computed from the corpus

Figure 1 presents the distribution of the cloze probability and surprisal of the most frequent response, the entropy of the sentences, the number of different responses, the number of invalid responses, and the reaction times. Regarding cloze probability, the distribution of the most probable sentence completions was nearly flat, indicating that sentence constraint was distributed across all possible cloze probability values. In particular, there were 965 (32.99%) sentences with a high cloze probability (> .70), 889 (30.39%) with medium (.40–.70), and 1071 (36.62%) with low (< .40). However, the distribution of surprisal values was left-skewed (*mean* = 1.07; *median* = 0.94; *range* = 0–4.06), suggesting that the responses contributed relatively little additional information to the overall sentence meaning. Furthermore, entropy was generally well distributed across all values (*mean* = 2.18; *median* = 2.22; *range* = 0– 5.24), but the highest entropy values were underrepresented. Thus, the corpus showed considerable variability in terms of the distribution of response probabilities within each sentence.

To obtain a more detailed description of the dataset, we performed Pearson's correlations among the following measures: a) cloze probability of the most frequent response, b) surprisal of the most frequent response, c) reaction times of the most frequent response, d) the number of different responses, and e) the number of invalid responses. As expected, all measures were significantly correlated (*r*_s_* >* |0.32|, *p*_s_* <* 0.001). The correlation among cloze probability, surprisal, and entropy was particularly high (*r*_s_* >* |0.94|, *p*_s_* <* 0.001), which is anticipated as cloze probability is used in the calculation of surprisal and entropy. Moreover, higher cloze probability, lower surprisal, and lower entropy resulted in fewer invalid responses and shorter reaction times (*r*_s_* >* |0.32|, *p*_s_* <* 0.001).

### Demographic exploration

Our investigation into demographic factors revealed interesting findings. When comparing responses by gender, we found no significant differences in cloze probability (*F*(1,5847) = 2.71, *p =* 0.09, *η*_*p*_^*2*^ < 0.001) or surprisal values (*F*(1,5847) = 2.75, *p =* 0.09, *η*_*p*_^*2*^ < 0.001) between women and men. However, men demonstrated lower entropy than women (*F*(1,5847) = 4.48, *p =* 0.034, *η*_*p*_^*2*^ = 0.001), suggesting that men had less variability in their responses. Additionally, men responded faster than women (*F*(1,5847) = 8.92, *p =* 0.003, *η*_*p*_^*2*^ = 0.002).

In terms of regional differences, there were no significant differences in cloze probability (*F*(1,5847) = 0.45, *p =* 0.49, *η*_*p*_^*2*^ < 0.001), surprisal (*F*(1,5847) = 0.51, *p =* 0.47, *η*_*p*_^*2*^ < 0.001), entropy (*F*(1,5847) = 0.75, *p =* 0.38, *η*_*p*_^*2*^ < 0.001), or reaction times (*F*(1,5847) = 0.35, *p =* 0.55, *η*_*p*_^*2*^ < 0.001) between those who lived in south-central Mexico (mainly Mexico City and the State of Mexico) and those from all other regions of Mexico.

Educational attainment showed no differences in cloze probability (*F*(1,5847) = 0.71, *p =* 0.39, *η*_*p*_^*2*^ < 0.001), surprisal (*F*(1,5847) = 1.53, *p =* 0.21, *η*_*p*_^*2*^ < 0.001), or entropy (*F*(1,5847) = 0.76, *p =* 0.38, *η*_*p*_^*2*^ < 0.001) between undergraduate and graduate participants. However, undergraduate students responded more slowly than their graduate counterparts (*F*(1,5847) = 12.87, *p* < 0.001, *η*_*p*_^*2*^ = 0.002). Similarly, the field of study seemed to impact only reaction times (*F*(1,5847) = 5.53, *p =* 0.01, *η*_*p*_^*2*^ = 0.001), with no impact on cloze probability (*F*(1,5847) = 0.16, *p =* 0.68, *η*_*p*_^*2*^ = 0.001), surprisal (*F*(1,5847) = 0.24, *p =* 0.62, *η*_*p*_^*2*^ < 0.001), or entropy (*F*(1,5847) = 0.18, *p =* 0.67, *η*_*p*_^*2*^ < 0.001); students from social science and humanities disciplines responded more quickly than students from other fields.

Exploring the neuropsychological profiles of our participants revealed that this factor only influenced reaction times (*F*(1,5847) = 10.008, *p =* 0.002, *η*_*p*_^*2*^ = 0.002). Neurotypical individuals responded more slowly than neuroatypical participants. However, no significant differences were observed in cloze probability (*F*(1,5847) = 0.45, *p =* 0.501, *η*_*p*_^*2*^ < 0.001), surprisal (*F*(1,5847) = 0.87, *p =* 0.34, *ηp*^*2*^ < 0.001), or entropy (*F*(1,5847) = 0.27, *p =* 0.601, *η*_*p*_^*2*^ < 0.001) between these groups.

Lastly, we examined the influence of language knowledge. Participants with proficiency in a language other than Spanish responded more quickly than those who identified themselves as monolinguals (*F*(1,5847) = 224.65, *p* < 0.001, *η*_*p*_^*2*^ = 0.037). However, there were no significant differences in cloze probability (*F*(1,5847) = 2.51, *p =* 0.11, *η*_*p*_^*2*^ < 0.001), surprisal (*F*(1,5847) = 2.17, *p =* 0.14, *η*_*p*_^*2*^ < 0.001), and entropy values (*F*(1,5847) = 1.03, *p =* 0.309, *η*_*p*_^*2*^ < 0.001) between multilingual and monolingual participants.

#### Norms comparisons

We carried out a developmental comparison between Mexican Spanish-speaking children and adults, selecting the 30 sentences with similar meaning and context for the analysis. The cloze probabilities for children's sentences were marginally higher (*M* = 0.76, *SD* = 0.19) than those of adults (*M* = 0.74, *SD* = 0.23). However, the observed difference was not statistically significant (*t*(29) = 0.75, *p =* 0.45).

For the dialectal comparison, we evaluated 45 sentences from Spanish speakers in Spain and Mexico. The results showed that Spanish participants from Spain (*M* = 0.55, *SD* = 0.33) provided a slightly higher cloze probability than their Mexican counterparts (*M* = 0.54, SD = 0.26). However, this difference was not statistically significant either (*t*(44) = 0.12, *p =* 0.90).

Finally, we compared language norms between other languages and our Spanish norms. Analyzing 1525 sentences, we found no significant differences (*t*(1524) = 0.46, *p =* 0.64) between the cloze probabilities of the non-Spanish language norms (*M* = 0.56, *SD* = 0.34) and our Spanish norms (*M* = 0.56, *SD* = 0.26).

## Discussion

This study provides sentence-completion norms in Mexican Spanish, covering 2925 sentence contexts evaluated by an average of 55 adults per context (mean age = 25 years). These norms present an invaluable resource for researchers, allowing them to select from a broad array of experimental stimuli for various fields of study, such as psycholinguistics, cognitive semantics, knowledge representation, grounded cognition, and computational linguistics, among others. The full corpus is accessible through the Open Science Framework at: https://osf.io/js359/?view_only=bb1b328d37d643df903ed69bb2405ac0.

The norms developed in this work span a wide range of values in terms of cloze probability, surprisal, and entropy, representing the varying degrees of sentence constraint. Consequently, the corpus enables researchers to select sentences with different constraint levels for sentence-completion tasks. In addition, the sentence contexts were characterized by their syntactic complexity and word morphology, allowing the selection of specific sentence types (e.g., coordinate vs. subordinate) for experimental purposes, to explore their behavioral effects and interactions with sentence constraint.

This work includes three different databases for calculating the constraint measure: the raw, corrected, and unified databases. This triple-tiered approach provides researchers with flexibility in deciding how the constraint measures should be computed. Researchers can use the provided MATLAB code to calculate all the measures detailed in this study. The raw database contains unedited responses, providing researchers with the freedom to develop their coding processes. This raw database can also be used to investigate specific questions, such as the types of errors that arise based on sentence constraints or syntactic structure.

The corrected database includes orthographic corrections and basic unification and cleaning procedures (e.g., standardizing all responses to lowercase, removing unnecessary spaces, and correcting typos). This database allows researchers to create their own criteria for unifying the responses. For instance, they may decide to use morphological inflections to examine the role of grammatical gender in relation to the level of constraint. This might involve calculating constraint measures for word endings of different grammatical genders, to measure the probability of a sentence providing a word of a particular gender.

Lastly, the unified database, while maintaining the correction and cleaning criteria of the corrected database (described above), adds more unification operations. This database focuses on constraints toward the concept, sidestepping lexical choice and morphological inflections. Thus, synonyms (e.g., *carro* or *coche* [*car*]), and gender and number, were unified according to their frequency and syntactic congruency with the sentence context. This unification allows for a numerical analysis of similar responses and it is primarily intended for psycholinguistic studies with research questions directed toward lexical access. However, it may not be as useful for specific inquiries into syntax or morphology.

The analysis presented in this study was conducted on unified responses, and it is possible that variations may occur if different coding criteria are applied. Nonetheless, the primary goal of achieving high variability in constraint measures was reached. Although the norms do not perfectly represent a flat distribution across all cloze probability values, with sentences having cloze probabilities in the .6 to .8 range being slightly underrepresented, there are still about 750 sentences within that range available for stimulus selection. This quantity should be more than adequate for most behavioral or physiological studies.

Our correlation analysis showed that all measures in the corpus behaved as expected: Constraint measures are highly correlated, which is anticipated since entropy and surprise are computed from cloze probability (refer to the Data analysis section). Importantly, the constraint of the sentence modulated reaction times and the number of valid and invalid responses. The higher constraint was associated with faster reaction times and fewer valid and invalid responses. This result aligns with lexical retrieval theories, suggesting that high-constraint sentences yield more specific lexical activation with less competition, thereby increasing processing speed during retrieval (Elman et al., 2004). Conversely, lower constraint sentences yield more general word activations, creating a more competitive environment and increasing the likelihood of retrieval failure. It is also worth considering how participants' demographic characteristics could influence the constraint of sentences. Interestingly, most previous norms do not report on whether these demographic characteristics were explored. We examined several relevant variables that could potentially modulate participants' responses, thereby increasing response variability and consequently reducing constraint levels.

With the demographic data, researchers have the option of excluding specific participants. However, is this a prudent course of action? To provide some perspective on this, we compared the cloze probability to demographic variables including gender, region of residence, education level, field of study, neuropsychological profile, and knowledge of a second language. Contrary to the theoretical expectations, most of these variables primarily influenced reaction times but did not significantly affect sentence constraint. Thus, if researchers are primarily interested in sentence constraint, they can use the measures computed over all participants.

The only variable that impacted one of the constraint measures was gender. Men exhibited higher entropy than women, suggesting that men produced responses with a greater distribution of probabilities. This outcome is not surprising as women typically possess larger lexicons (Hyde & Linn, 1988), enabling them to select more specific lexical candidates. This increased competition could explain why women responded slower than men, although it is worth noting that men usually respond faster than women in motor tasks (Bianco et al., 2020) and exhibit higher levels of impulsivity (Ramos-Loyo et al., 2016).

Remarkably, we found no significant differences in any measures between participants from different regions of Mexico. This finding implies that our sentences are reliable for measuring psycholinguistic processes regardless of the dialectal variant of Mexican Spanish spoken by the participant. This is not surprising since Mexican Spanish is similar in all the country while still showing more or less marked lexical differences in their different geographic regions (Lope Blanch, 2004).

Participants with higher educational backgrounds, especially those from social sciences and humanities, responded more quickly. This outcome likely reflects their familiarity with written language (Hernández Muñoz, 2015). Higher education often fosters an environment that promotes quick thinking, problem-solving, and complex reasoning. These cognitive challenges may enhance neural connectivity and improve overall brain function, leading to faster reaction times. Furthermore, social science disciplines such as sociology, anthropology, and psychology place a significant emphasis on effective communication, which involves rapidly understanding, interpreting, and responding to linguistic cues. This skillset might facilitate quicker responses to language tasks.

We included a small subset of participants who reported neuroatypical profiles, such as neurodevelopmental, psychiatric, and neurological disorders. The prevalence of these conditions in our participant pool was consistent with the general population (American Psychiatric Association, 2013). Interestingly, participants who identified as neuroatypical responded more quickly than those who identified as neurotypical. This phenomenon could potentially be explained by the fact that various neuroatypical profiles share impulsivity as a common symptom (Kulacaoglu & Kose, 2017). However, given the small number of participants and the variety of neuropsychological profiles, this conclusion should be interpreted cautiously. For instance, individuals with certain neurodevelopmental conditions such as dyslexia typically read slower than neurotypical individuals (American Psychiatric Association, 2013).

Lastly, participants who reported knowledge of a second language responded faster than those who were identified as monolingual. Previous research showed that the decreased reaction times observed in bilinguals during language tasks are often attributed to their enhanced cognitive control and executive functions. This is because managing two or more languages necessitates certain mental skills, such as attention control, inhibitory control, and mental flexibility (Bialystok, 2015). Even though our participants are not highly skilled in two languages, they still show a benefit in our task.

Regarding the norms comparisons, we observed an unexpected outcome: despite differences in developmental stage, dialectal variant, or language, the sentences adapted from other norms yielded similar scores to those evaluated in the present study. This result contrasts with previous research documenting such effects (Arcuri et al., 2001; Rossi et al., 2020). A possible explanation is that the contexts adapted from other norms were relatively general (e.g., "in the airport she boards the"), thereby requiring participants to use general knowledge and frequently occurring words to complete the task. As a result, while participants responded with different words, the constraint remained consistent. It is worth noting that studies reporting differences in cloze probability between dialectal variants did not incorporate cultural adaptations of the sentences (Arcuri et al., 2001). In contrast, studies, where cultural adaptations were performed, found similar results regarding cloze probability (Lahar et al., 2004). This research indeed brings attention to the intriguing issue of cultural adaptation in sentence comprehension. On the surface, it appears that assessing sentences derived from other cultures may alter the responses. However, it becomes evident that when the underlying meaning of the structure is adequately adapted to the culture in question, participants across cultures behave similarly. This suggests that despite potential cultural or dialectal differences in language usage, the essential meaning of sentences remains generally consistent across diverse cultural contexts. The implication of this observation could be significant, especially for future psycholinguistic research that seeks to compare and understand language comprehension across different cultural or linguistic groups.

Concerning developmental differences, we did not replicate prior research indicating an increase in cloze probability with age Lahar et al. (2004). We must acknowledge that our subsample of sentences was likely underpowered as we only included 30 sentences from the norms for Mexican children. However, our primary explanation is that these sentences predominantly represented medium and high cloze probability. The most striking developmental change in these kinds of tasks is a reduction in the number of responses and invalid responses (Lahar et al., 2004; Pinheiro et al., 2010; Rossi et al., 2020). This effect primarily influences contexts with lower constraint since highly constraining sentences usually have only one viable answer. Consequently, the sentences selected for the children's norms would not be significantly impacted by this developmental effect.

## Considerations

Firstly, note that while our sample size of around 55 participants is larger than the average usually seen in many psycholinguistic studies (around 30, e.g., DeLong et al., 2005), the demographic characteristics of the participants are not evenly distributed across all sentences. For instance, participants who are native Spanish speakers and also have knowledge of a second language constitute 50% of the sample. Hence, removing them would reduce the average number of participants per sentence to approximately 25. This reduction would affect the variability of responses, and subsequently, the entropy measure. However, measures such as cloze probability or surprisal might be less impacted. The reduction in sample size could also have a more significant effect on sentences with medium and lower constraints.

Secondly, it should be acknowledged that this corpus was primarily evaluated by young students from Mexico. Thus, caution should be taken when extrapolating these responses to other populations with differing demographic characteristics, such as children or illiterate individuals.

Thirdly, although the expected negative correlation between reaction times and cloze probability values was observed, it should be highlighted that these measures were obtained using a variety of devices such as mobile phones, tablets, and computers. This is a factor that should be taken into account when interpreting the results. Nevertheless, measuring reaction times with JavaScript carries certain limitations because the time needed to process an event depends on factors like the participant's computer, the browser they are using, and other tasks the computer is executing at the time. However, response time measurements in jsPsych and in general web-based HTML/JavaScript have been found to be comparable to standard lab software like MATLAB's Psychophysics Toolbox and E-Prime (de Leeuw & Motz, 2016; Hilbig, 2016).

Recent evaluations have gauged the precision of reaction time measurements across different packages for running online experiments (such as jsPsych, PsychoPy, Testable, Lab.js, Gorilla), operating systems (like Ubuntu, macOS, Windows 10), and browsers (such as Chrome, Firefox) (Bridges et al., 2020). While these online experimental packages did not perform as well as their lab-based counterparts, the results were still surprisingly good. Pertinently for this study, the reaction times measured using jsPsych had a variability of 5 to 10 ms (standard deviation) across the different operating systems and browsers (Bridges et al., 2020). Moreover, the use of longer reaction times (as in this experiment, given the time needed to write the complete word) helps to mitigate the variability caused by computer hardware and software in between-subjects designs (de Leeuw, 2015).

## Conclusions

The research presented here provides sentence-completion norms for Mexican Spanish, comprising of 2925 sentence contexts, each evaluated by around 55 adults. This comprehensive resource has been made available for researchers in several fields, including psycholinguistics, cognitive semantics, and computational linguistics, and is designed to aid in the selection of experimental stimuli. The norms offer a wide range of cloze probability, surprisal, and entropy values, which indicate the degree of sentence constraint. Additionally, the sentence contexts were characterized based on syntactic complexity and word morphology. The results demonstrated that the constraint measures were highly correlated, and sentence constraint was found to modulate reaction times and the number of valid and invalid responses. Interestingly, demographic variables were found to influence reaction times but not sentence constraint. Importantly, the norms can be used to support further research in diverse areas and contexts and can be adapted to specific research questions, thereby expanding our understanding of language processing and other related fields. As such, this work provides a scaffold towards more nuanced and comprehensive studies in the future.

## Data Availability

All data and codes are freely available in the Open Science Framework repository at: https://osf.io/js359/?view_only=bb1b328d37d643df903ed69bb2405ac0

## Appendix

The instructions were presented on four individual pages to facilitate comprehension. Participants needed to press the Continue button to complete reading the instructions, and they could press the Back button if they needed to revise again the previous instruction. Note that the original language of instructions was Spanish.

### Instructions

(P. 1/4) In the activity you will carry out, you will be presented with a series of sentences that are missing the last word. Your task is to write the word with which you think the sentence is best completed. It is important that you write the first word you thought of to complete the sentence as quickly as possible. You have 15 s to give your answer. If you don't type the word within 15 s, the next sentence will be presented automatically.

(P. 2/4) There are no correct or incorrect answers. You should not worry about how the word you think is spelled. Spelling is not important for this activity.

(P. 3/4) This is an example sentence just as they will appear in the task:

A. You will be presented with a sentence that is missing the last word.
B. Write the first word that you think best completes the sentence. Do not write more than one word.
C. To continue press the "ENTER" key on the keyboard or touch the "ENTER" button if you are using a touch screen.

(P. 4/4) You can check your completion progress of the activity by the bar that appears at the top of the screen. If you want to read the instructions again, press the Back button. Instead, press the Continue button to do some practice sentences.
